# Demographic and surgical characteristics in patients who do not achieve minimal important change in the KOOS Sport/Rec and QoL after ACL reconstruction: a comparative study from the Swedish National Knee Ligament Registry

**DOI:** 10.1136/bmjopen-2023-083803

**Published:** 2024-09-05

**Authors:** Rebecca Simonsson, Judy Bittar, Janina Kaarre, Bálint Zsidai, Mikael Sansone, Ramana Piussi, Volker Musahl, James Irrgang, Kristian Samuelsson, Eric Hamrin Senorski

**Affiliations:** 1 University of Gothenburg, Goteborg, Sweden; 2 Department of Orthopaedics, University of Gothenburg, Goteborg, Sweden; 3 Department of Orthopaedics, Medical sciences, Goteborg, Sweden; 4 Ortopedi, Sahlgrenska Academy, Goteborg, Sweden; 5 Sahlgrenska Sports Medicine Center, Gothenburg, Sweden; 6 Department of Orthopaedic Surgery, University of Pittsburgh, Pittsburgh, Pennsylvania, USA; 7 University of Pittsburgh, Pittsburgh, Pennsylvania, USA; 8 Sahlgrenska University Hospital, Gothenburg, Sweden; 9 Neuroscience and physiology, Health and rehabilitation, Goteborg, Sweden

**Keywords:** Knee, SPORTS MEDICINE, Musculoskeletal disorders, Orthopaedic sports trauma, REGISTRIES, Psychometrics

## Abstract

**Abstract:**

**Objectives:**

This study aimed to compare demographic and surgical characteristics between patients who do and do not achieve minimal important change (MIC) in the Knee injury and Osteoarthritis Outcome Score (KOOS) Sports and Recreation (Sport/Rec) and Quality of Life (QoL) subscales 1 year after anterior cruciate ligament reconstruction.

**Design:**

Comparative cross-sectional.

**Setting:**

The MIC for the KOOS Sport/Rec subscale was ≥12.1 and ≥18.3 for the KOOS QoL subscale from before surgery to 1-year follow-up using data from the Swedish National Knee Ligament Registry.

**Participants:**

In total 16 131 patients were included: 11 172 (69%) with no MIC for the Sport/Rec scale, and 10 641 (66%) for the QoL.

**Results:**

Patients with no MIC for Sport/Rec and QoL had a higher body mass index (BMI) (24.8±3.5 vs 24.6±3.3 and 24.7±3.5 vs 24.6±3.2, respectively, p<0.0001), were younger (years) at time of surgery (28.5±10.3 vs 29.1±10.8 and 27.4±9.8 vs 29.7±11.0, respectively, p=0.0002 and <0.0001), had longer time from injury to surgery (months) (Sports/Rec 22.0±38.5 vs 19.3±36.6, respectively, p=0.0002), and greater rates of concomitant cartilage injuries especially to the lateral femoral condyle (22.7% vs 19.4% and 23.3% vs 19.0%, respectively, p=0.001 and p=0.005) compared with patients who achieved the MIC. A smaller proportion of patients treated with a hamstring tendon autograft had no MIC (91.4%) compared with patients with MIC (94.1%).

**Conclusions:**

Patients with no MIC for KOOS Sport/Rec and QoL subscales had a higher BMI, longer time from injury to surgery and were younger at the time of surgery compared with patients who did achieve MIC. Although differences were small, they may reframe management strategies with patients who have these characteristics.

**Strengths and limitations of this study**

- This is a registry-based study, enabling a large sample size.
- A large sample size allows a comprehensive capture of the study group.
- Using data from the registry may limit generalisability, since data only provide information about the Swedish Knee Ligament Registry population with anterior cruciate ligament reconstruction.
- The minimal important change can be calculated in different ways to match different data sets, and might not apply to every unique patient.

## Introduction

 Despite good surgical outcomes in terms of recovery of knee function after anterior cruciate ligament reconstruction (ACLR), a subset of patients continues to experience suboptimal knee function, with a subsequent negative impact on quality of life and ability to continue with sports and recreational activities.^1^ Thus, patient-reported outcomes (PROs) have been frequently used to assess the patient’s perception of their knee function and to evaluate the effects and outcomes of surgical treatment of an ACL injury.^2^,^4^ One PRO commonly used in patients treated with ACLR is the Knee injury and Osteoarthritis Outcome Score (KOOS), which contains five subscales which evaluate pain, symptoms, activities of daily living (ADL), sport and recreation, and quality of life.^5^ However, only the KOOS subscales sports and recreation, and quality of life fulfilled the criteria of unidimensionality for patients treated with ACLR.^6^ Despite a wide range of studies that report outcomes for patients with an ACL injury with PROs,^7^ interpretation of the scores is still difficult for the ACL research. To aid clinicians in interpreting scores from PROs, several concepts can be calculated, for example, the minimum clinically important difference (MCID) and the minimal important change (MIC). The MCID represents the smallest improvement considered worthwhile by a patient.^8^ The MIC instead reflects the smallest measured change in score that patients perceive as important.^9^ When it comes to clinically meaningful effects, the terms MIC and MCID are used, not always consistently, and often in ways that make interpretation difficult.^10^ The MIC and the MCID are fundamentally different.^10^ As semantically described, a change (MIC) occurs within one group, while a difference (MCID) occurs between groups.^11^

Although numerous factors have been reported to affect outcomes after ACLR including graft failure,^12 13^ reinjury^14 15^ and inferior PROs,^16 17^ factors associated with clinically relevant changes in PROs remain to a large extent undefined. Importantly, patient demographics and surgical factors, have previously been shown to affect patient-reported knee function as measured with the KOOS subscales.^18^,^20^ However, current evidence is limited regarding the possible demographical and surgical differences in patients who achieve a MIC in the KOOS subscales sports and recreation and quality of life following ACLR. In addition, a deeper understanding of which patients are not likely to experience clinical improvement after ACLR and do thereby not respond to ACLR as a treatment is of evident need in order for patients to receive the best possible treatment after ACL injury.

The aim of this study was to compare demographic and surgical characteristics recorded at the time of surgery between patients who do and do not achieve a MIC for the KOOS Sports and Recreation (Sport/Rec) and Quality of Life (QoL) subscales 1 year after ACLR.

## Methods

### Study design

This study was conducted as a comparative registry study, where data was analysed cross-sectionally and was reported according to the Reporting of studies Conducted using Observational Routinely collected Data statement,^21^ which is an extension of the Strengthening the Reporting of Observational Studies in Epidemiology guidelines^22^ with data from the Swedish National Knee Ligament Registry (SNKLR) during the period January 2005–May 2019.

The SNKLR was established in 2005, and covers over 90% of all ACLRs performed in Sweden every year.^23^ The SNKLR aims to promote superior treatment for individuals with ACL injuries by providing hospitals and clinics with accurate feedback with regard to surgical technique and outcomes after ACLR, and to determine prognostic factors for successful and poor outcomes.^24^ The SNKLR has two different sources of information. The primary source is registered by a medical professional, and contains information on demographics, injury-related factors and treatment-specific data for patients undergoing surgical and non-surgical treatment after an ACL injury. The secondary source contains information registered by the patient including PROs,^24^ including the KOOS^5^ and EuroQoL-5 Dimension.^25^ Only the KOOS was used in this study.

Data from both sections are registered in the SNKLR at baseline, which corresponds to the time of surgical treatment. Patients are asked to respond to questionnaires at 1-, 2-, 5- and 10-year follow-ups after ACLR.

Activity at the time of injury is registered by choosing one activity from predefined answer alternatives, which is then registered to the individual patient. The activities at the time of injury presented in this study are grouped based on type of activity, that is, pivoting sports (soccer, handball, basketball, American football, rugby, dancing, floorball, gymnastics, ice hockey, bandy, martial arts, racket sports, volleyball and wrestling), alpine skiing, non-pivoting sports (cross-country skiing, cycling, motocross/enduro, snowboarding, surfing, wakeboarding, horseback riding, skateboarding), other physical activity (other recreational sports), traffic-related injuries and other (outdoor and walking).

### Study population

Patients eligible for inclusion were ≥15 years old, had undergone a primary ACLR and had KOOS data for the Sport/Rec and QoL subscales before surgery and at 1-year follow-up. Patients with previous knee surgery, associated fracture to the patella, femur, tibia or fibula, or concomitant nerve, vascular or additional injuries to any other knee ligaments, such as the posterior cruciate ligament, or had a surgically reconstructed medial or lateral collateral ligament (MCL, LCL) tear were excluded from this study. In addition, patients with high KOOS at baseline for Sport/Rec (>87.9) and for QoL (>81.7),^26^ and thereby not able to achieve the MIC, were excluded from this study. Data regarding patients (age, sex, body mass index (BMI) and activity at the time of injury), and surgical characteristics (graft type (hamstring-, patellar- or quadriceps tendon autograft, allograft) and associated injuries) were obtained from the registry. Included patients were divided into two groups, (1) the MIC group, consisting of patients who did achieve the MIC and (2) no MIC group, patients who did not achieve MIC for one or both of the KOOS subscales Sport/Rec and QoL, between preoperative values and 1-year follow-up.

### Outcome measure

The main outcome of interest in this study was differences in demographical (eg, age, sex, BMI, activity at time of injury) and surgical factors (eg, type of graft, cartilage injuries, meniscal injuries, collateral ligament injuries) between patients who achieved the MIC and patients who did not achieve the MIC, for the KOOS subscales Sport/Rec and QoL, between preoperative ratings and 1 year after ACLR. The MIC published by Ingelsrud *et al*^26^ of ≥12.1 for Sport/Rec and ≥18.3 for QoL was used in this study. The MIC reflects the smallest measured change in score that patients perceive as being important, that is, the patient’s function has improved by treatment.^27^ The MIC refers to the change from baseline to a specific follow-up timepoint^10^ for a given PRO that is perceived as being important by a patient. The MIC used in this study was calculated using an anchor-based method and predictive modelling, as this method anchors the PRO change score to an external measure of important change.^26^ Furthermore, the anchor-based method has the best accuracy in predictive modelling and does not easily overestimate the MIC.

The KOOS was developed in 1998 for the assessment of an individual’s precepted knee function in patients with knee-related conditions.^28^ The KOOS aims to assess symptoms and functional limitations following a knee injury and consists of 42 items scored on five subscales: Pain, Symptoms, Function in Daily Living (ADL), Sport/Rec and QoL.^29^ All items are scored using a Likert scale, with five response options ranging from 0 meaning extremely positive to 4 meaning extremely negative response. Scores are then converted to a 0–100 scale, where 0 represents the worst possible knee function and 100 suggests the best possible knee function. The KOOS has interclass correlation coefficients that range from 0.85 to 0.90 for the five subscales,^30^ however, there is no evidence for the content validity of the KOOS.^7^

### Patient and public involvement

Patients and/or the public were not involved in the design, or conduct, or reporting, or dissemination plans of this research.

### Statistics

Statistical analyses were performed with SAS Statistics for Windows (V.9.4, SAS Institute, Cary, NC, USA). For comparison between groups, Fisher’s exact test and χ^2^ test were used for categorical variables, while the Mann-Whitney U test was used for continuous variables. Comparative analyses were performed for demographic and surgical characteristics between patients who achieved MIC and patients who did not, from baseline to 1-year follow-up for both the KOOS Sport/Rec and QoL. Continuous variables were presented as mean and SD as well as median with minimum and maximum. Count (n) and proportion (%) were used for presenting categorical variables. The significance level was set at p<0.05. Logistic regression was used to adjust for differences in age at surgery and preoperative KOOS. Adjustment for age at surgery was made as patient outcomes differ depending on age, and surgical methods can differ depending on age. Adjustment for preoperative KOOS was made as scores can differ in terms of how easy it can be to achieve MIC, that is, the greater score preinjury, the more difficult to achieve MIC postinjury. Missing data were defined as ‘missing’ in actual tables.

## Results

**Figure 1:**
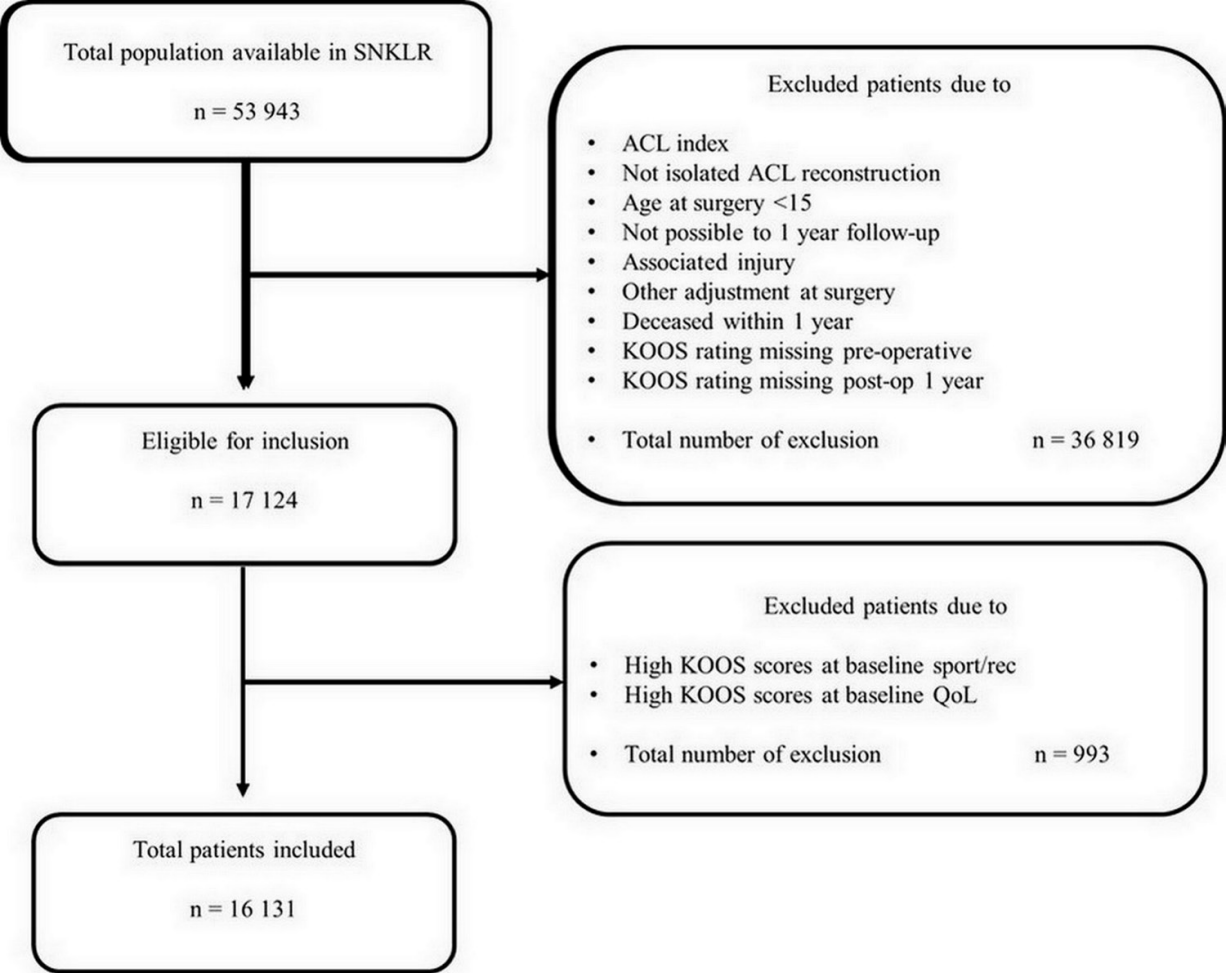
Flowchart of the selection process. ACL, anterior cruciate ligament; KOOS, Knee injury and Osteoarthritis Outcome Score; QoL, Quality of Life; SNKLR, Swedish National Knee Ligament Registry; Sport/Rec, Sports and Recreation.

A total of 16 131 patients were included in the study, of which 11 172 (69%) did not achieve the MIC for the Sport/Rec and 10 641 (66%) for the QoL subscales. The selection process up to inclusion is presented in figure 1.

### Demographic differences and the KOOS Sport/Rec

Table 1 presents results for the KOOS Sport/Rec both for demographics and concomitant injuries. A higher number of male patients (51.5%) achieved MIC, while a higher number of female patients did not achieve MIC (49.2%) for KOOS Sport/Rec (p<0.0001). The no MIC group had a significantly greater BMI compared with the MIC group (24.8±3.5 vs 24.6±3.3, respectively, p<0.0001). A greater proportion of patients in the no MIC group were younger at the time of surgery (28.5±10.3 vs 29.1±10.8 years, respectively, p=0.0002) and had a longer time from injury to surgery (22.0±38.5 vs 19.3±36.6 months, respectively, p=0.0002) compared with patients in the MIC group. Furthermore, there was a smaller proportion of patients treated with a hamstring tendon autograft in the no MIC group (91.4%) compared with the MIC group (94.1%). On the other hand, there was a greater proportion of patients treated with patellar tendon autograft in the no MIC group (6.3%) (p=0.0023) compared with the MIC group (4.2%).

**Table 1:** Demographics between patients who achieve MIC and patients who do not achieve MIC in KOOS Sports and Recreation

Variable	Total	MIC	No MIC	P value	Adjusted p value*	Difference between groups mean (95% CI)
n	16 131	11 172	4959			
Female, n (%)	7857 (48.7%)	5419 (48.5%)	2438 (49.2%)	0.45	<0.0001	−0.7 (−2.3 to 1.0)
Male, n (%)	8274 (51.3%)	5753 (51.5%)	2521 (50.8%)			0.7 (−1.0 to 2.3)
BMI (kg/m^2^)	24.6 (3.3)	24.6 (3.3)	24.8 (3.5)	0.04	<0.00001	−0.2 (−0.3 to −0.04)
	24.2 (15.1; 45.4)	24.2 (15.1; 44.8)	24.3 (16; 45.4)			
	n=11 641	n=8233	n=3408			
Age at surgery (years)	28.9 (10.7)	29.1 (10.8)	28.5 (10.3)	0.02	0.0002	0.6 (0.3 to 0.9)
	26 (16–74)	26 (16–74)	26 (16–71)			
	n=16 131	n=11 172	n=4959			
Time from injury to surgery (months)	20.1 (37.2)	19.3 (36.6)	22.0 (38.5)	<0.0001	0.0002	−2.7 (−4.0 to −1.4)
	7.9 (0–551)	7.4 (0–456.5)	9.1 (0.1–551)			
	n=15 749	n=10 913	n=4836			
ACL tendon autograft						
Patella	768 (4.8%)	460 (4.2%)	308 (6.3%)	<0.0001	0.002	
Hamstring	14 835 (93.3%)	10 377 (94.1%)	4458 (91.4%)			
Quadriceps	237 (1.5%)	149 (1.4%)	88 (1.8%)			
Allograft	32 (0.2%)	18 (0.2%)	14 (0.3%)			
Other	32 (0.2%)	24 (0.2%)	8 (0.2%)			
Missing	227	144	83			
Injury						
Concomitant injury	9240 (57.3%)	6463 (57.8%)	2777 (56.0%)	0.03	0.08	−1.9 (−3.5 to −0.2)
Meniscal injury (medial and/or lateral)	7086 (43.9%)	5037 (45.1%)	2049 (41.3%)	<0.0001	0.08	−3.8 (−5.4 to −2.1)
Lateral menisci	4016 (24.9%)	2857 (25.6%)	1159 (23.4%)	0.003	0.09	−2.2 (−3.6 to −0.8)
Medial menisci	4234 (26.2%)	3022 (27.0%)	1212 (24.4%)	0.0005	0.67	−2.6 (−4.1 to −1.1)
Lateral and medial menisci	1164 (7.2%)	842 (7.5%)	322 (6.5%)	0.02	0.92	−1.0 (−1.9 to −0.2)
Cartilage	4356 (27.0%)	2980 (26.7%)	1376 (27.7%)	0.16	<0.0001	1.1 (−0.4 to 2.6)
LFC	886 (20.4%)	574 (19.4%)	312 (22.7%)	0.01	0.001	3.4 (0.7 to 6.1)
MFC	2932 (67.6%)	2016 (68.0%)	916 (66.8%)	0.43	0.74	−1.3 (−4.3 to 1.8)
PL	537 (12.4%)	351 (11.8%)	186 (13.6%)	0.12	0.02	1.7 (−0.5 to 3.9)
PM	871 (20.1%)	590 (19.9%)	281 (20.5%)	0.69	0.09	0.6 (−2.1 to 3.2)
LTP	1083 (25.0%)	718 (24.2%)	365 (26.6%)	0.10	0.04	2.4 (−0.5 to 5.2)
MTP	826 (19.0%)	566 (19.1%)	260 (19.0%)	0.95	0.23	−0.1 (−2.7 to 2.4)
Trochlea	530 (12.2%)	362 (12.2%)	168 (12.2%)	1.00	0.25	0.0 (−2.1 to 2.2)
Multicartilage	4336 (26.9%)	2964 (26.5%)	1372 (27.7%)	0.14	<0.0001	1.1 (−0.4 to 2.6)
LCL	107 (0.7%)	72 (0.6%)	35 (0.7%)	0.73	0.29	0.1 (−0.2 to 0.4)
MCL	562 (3.5%)	398 (3.6%)	164 (3.3%)	0.44	0.52	−0.3 (−0.9 to 0.4)
PLC	19 (0.1%)	11 (0.1%)	8 (0.2%)	0.40	0.16	0.1 (−0.1 to 0.2)
Multiligamentous	21 (0.1%)	12 (0.1%)	9 (0.2%)	0.33	0.06	0.1 (−0.1 to 0.2)

For categorical variables n (%) is presented. For continuous variables mean (SD)/median (min; max)/(95% CI for mean)/n=is presented. For comparison between groups Fisher’s exact test (lowest one-sided p value multiplied by 2) was used for dichotomous variables and the Mann-Whitney U test was used for continuous variables.

* Analysis adjusted for age at surgery and preoperative KOOS.

ACLanterior cruciate ligamentBMIbody mass indexKOOSKnee injury and Osteoarthritis Outcome ScoreLCLlateral collateral ligamentLFClateral femoral condyleLTPlateral tibia plateauMCLmedial collateral ligamentMFCmedial femoral condyleMICminimal important changeMTPmedial tibial plateaunnumberPLpatella lateralPLCposterior lateral cornerPMpatella medialQoLQuality of LifeSport/RecSports and Recreation

### Concomitant injuries and the KOOS Sport/Rec

Table 1 presents results for the KOOS Sport/Rec subscale both for demographics and concomitant injuries. Patients in the no MIC group had a greater rate of concomitant cartilage injuries (27.7% vs 26.7%, respectively, p<0.0001) especially located on the lateral femoral condyle (22.7% vs 19.4%, respectively, p=0.001) or in the presence of a multicartilage injury (27.7% vs 26.5%, p<0.0001) compared with patients in the MIC group.

### Preoperative scores for KOOS Sport/Rec

Table 2 presents preoperative and postoperative scores for the KOOS Sport/Rec subscale. Patients in the no MIC group had significantly greater scores preoperative (48.5±25.2) compared with the MIC group (31.6±22.6) p<0.0001. Patients in the no MIC group also had lowered their scores postoperatively and had significantly lower scores postoperative compared with the MIC group (48.2±26.5 vs 73.0±20.9, respectively, p<0.0001).

**Table 2:** Preoperative and postoperative scores for KOOS Sport/Rec

Variable	Total	MIC	No MIC	P value	Adjusted p value*	Difference between groups mean (95% CI)
n	16 131	11 172	4959			
Female	7857 (48.7%)	5419 (48.5%)	2438 (49.2%)	0.45	<0.0001	−0.7 (−2.3 to 1.0)
Male	8274 (51.3%)	5753 (51.5%)	2521 (50.8%)			0.7 (−1.0 to 2.3)
Preoperative	36.8 (24.7)	31.6 (22.6)	48.5 (25.2)	<0.0001	<0.0001	−16.9 (−17.7 to −16.1)
	35 (0–85)	30 (0–85)	50 (0–85)			
	n=16 131	n=11 172	n=4959			
Postoperative KOOS 1 year	63.7 (26.7)	73.0 (20.9)	42.8 (26.5)	<0.0001		30.1 (29.3 to 31.0)
	70 (0–100)	75 (15–100)	45 (0–95)			
	n=16 125	n=11 172	n=4953			

For categorical variables n (%) is presented. For continuous variables Mmean (SD) / Median (Mmin; Mmax) / (95% CI CI for Mmean) / n=is presented. For comparison between groups Fisher’´s Eexact test (lowest 1one-sided p- value multiplied by 2) was used for dichotomous variables and the Mann-Whitney U- test was used for continuous variables.

* Analysis adjusted for age at surgery and pre-operativepreoperative KOOS. score

KOOS, Knee injury and Osteoarthritis Outcome Score; MIC, minimal important change; n, number

### Demographic differences and the KOOS QoL

Table 3 presents results for the KOOS QoL subscale for demographics and concomitant injuries. Patients in the no MIC group for KOOS QoL had greater BMI (24.7±3.5 vs 24.6±3.2, respectively, p=0.0001) and were younger at the time of surgery compared with patients in the MIC group (27.4±9.8 vs 29.7±11.0, respectively, p<0.0001). There were no significant differences between patients in the no MIC or MIC group for the KOOS QoL subscale with regards to patient sex, time from injury to surgery, or type of autograft.

**Table 3:** Demographics between patients who achieve MIC and patients who does not achieve MIC in KOOS QoL

Variable	Total	MIC	No MIC	P value	Adjusted p value	Difference between groups mean (95% CI)
n	16 131	10 641	5490			
Female, n (%)	7857 (48.7%)	5240 (49.2%)	2617 (47.7%)	0.060	0.30	1.6 (−0.1 to 3.2)
Male, n (%)	8274 (51.3%)	5401 (50.8%)	2873 (52.3%)			−1.6 (−3.2 to 0.1)
BMI	24.6 (3.3)	24.6 (3.2)	24.7 (3.5)	0.33	0.0001	−0.01 (−0.1 to 0.1)
	24.2 (15.1–45.4)	24.2 (15.1–44.8)	24.2 (16–45.4)			
	n=11 641	n=7838	n=3803			
Age at surgery	28.9 (10.7)	29.7 (11.0)	27.4 (9.8)	<0.0001	<0.0001	2.3 (2.0 to 2.7)
	26 (16–74)	27 (16–74)	25 (16–71)			
	n=16 131	n=10 641	n=5490			
Time from injury to surgery (months)	20.1 (37.2)	20.5 (38.5)	19.4 (34.8)	0.01	0.08	1.1 (−0.1 to 2.3)
	7.9 (0–551)	7.8 (0–458.4)	8.2 (0.1–551)			
	n=15 749	n=10 360	n=5389			
ACL tendon autograft						
Patella	768 (4.8%)	482 (4.6%)	286 (5.3%)	0.17	0.49	
Hamstring	14 835 (93.3%)	9833 (93.6%)	5002 (92.6%)			
Quadriceps	237 (1.5%)	147 (1.4%)	90 (1.7%)			
Allograft	32 (0.2%)	19 (0.2%)	13 (0.2%)			
Other	32 (0.2%)	21 (0.2%)	11 (0.2%)			
Missing	227	139	88			
Injury						
Concomitant injury	9240 (57.3%)	6156 (57.9%)	3084 (56.2%)	0.04	0.02	−1.7 (−3.3 to −0.0)
Meniscal injury (medial and/or lateral)	7086 (43.9%)	4755 (44.7%)	2331 (42.5%)	0.01	0.93	−2.2 (−3.9 to −0.6)
Lateral menisci	4016 (24.9%)	2653 (24.9%)	1363 (24.8%)	0.90	0.91	−0.1 (−1.5 to 1.3)
Medial menisci	4234 (26.2%)	2873 (27.0%)	1361 (24.8%)	0.003	0.31	−2.2 (−3.6 to −0.8)
Lateral and medial menisci	1164 (7.2%)	771 (7.2%)	393 (7.2%)	0.87	0.08	−0.1 (−0.9 to 0.8)
Cartilage	4356 (27.0%)	2924 (27.5%)	1432 (26.1%)	0.06	0.002	−1.4 (−2.8 to 0.1)
LFC	886 (20.4%)	554 (19.0%)	332 (23.3%)	0.001	0.01	4.2 (1.6 to 6.9)
MFC	2932 (67.6%)	1975 (67.9%)	957 (67.1%)	0.64	0.45	−0.8 (−3.8 to 2.3)
PL	537 (12.4%)	363 (12.5%)	174 (12.2%)	0.84	0.06	−0.3 (−2.4 to 1.9)
PM	871 (20.1%)	587 (20.2%)	284 (19.9%)	0.88	0.03	−0.3 (−2.8 to 2.3)
LTP	1083 (25.0%)	708 (24.3%)	375 (26.3%)	0.17	0.12	2.0 (−0.9 to 4.8)
MTP	826 (19.0%)	571 (19.6%)	255 (17.9%)	0.18	0.29	−1.7 (−4.2 to 0.8)
Trochlea	530 (12.2%)	365 (12.5%)	165 (11.6%)	0.39	0.11	−1.0 (−3.1 to 1.1)
Multicartilage	4336 (26.9%)	2910 (27.3%)	1426 (26.0%)	0.07	0.002	−1.4 (−2.8 to 0.1)
LCL	107 (0.7%)	75 (0.7%)	32 (0.6%)	0.43	0.96	−0.1 (−0.4 to 0.1)
MCL	562 (3.5%)	382 (3.6%)	180 (3.3%)	0.33	0.82	−0.3 (−0.9 to 0.3)
PLC	19 (0.1%)	9 (0.1%)	10 (0.2%)	0.15	0.02	0.1 (−0.0 to 0.2)
Multiligamentous	21 (0.1%)	14 (0.1%)	7 (0.1%)	1.00	0.37	−0.0 (−0.1 to 0.1)

For categorical variables n (%) is presented. For continuous variables mean (SD)/median (min; max)/(95% CI for mean)/n=is presented. For comparison between groups Fisher’s exact test (lowest one-sided p value multiplied by 2) was used for dichotomous variables and the Mann-Whitney U test was used for continuous variables.

* Analysis adjusted for age at surgery and preoperative KOOS.

ACLanterior cruciate ligamentBMIbody mass indexKOOSKnee injury and Osteoarthritis Outcome ScoreLCLlateral collateral ligamentLFClateral femoral condyleLTPlateral tibia plateauMCLmedial collateral ligamentMFCmedial femoral condyleMICminimal important changeMTPmedial tibial plateaunnumberPLpatella lateralPLCposterior lateral cornerPMpatella medialQoLQuality of LifeSport/RecSports and Recreation

### Concomitant injuries and the KOOS QoL

Table 3 presents patients in the no MIC group who had a greater rate of concomitant cartilage injuries to the lateral femoral condyle (23.3% vs 19.0%, p=0.01), however, lower rate of general cartilage injury (26.1% vs 27.5%, respectively, p=0.002), to the medial patella (29.9% vs 20.5%, p=0.03) and multicartilage injuries (26.0% vs 27.3%, p=0.002) compared with patients in the MIC group.

### Preoperative scores for KOOS QoL

Table 4 presents the preoperative and postoperative scores for KOOS QoL. Patients in the no MIC group had significantly greater scores preoperative (35.4±17.4) compared with the MCI group (30.5±15.9), p<0.0001. Both patients in the no MIC and MIC group had increased their scores postoperatively, although patients in the no MIC group had significantly lower scores compared with the patients in the MIC group overall (43.1±21.4 vs 64.5±20.5, respectively, p<0.0001).

**Table 4:** Preoperative and postoperative scores for KOOS QoL scores

Variable	Total	MIC	No MIC	P value	Adjusted p value*	Difference between groups mean (95% CI)
n	16 131	11 172	4959			
Female	7857 (48.7%)	5419 (48.5%)	2438 (49.2%)	0.45	<0.0001	−0.7 (−2.3 to 1.0)
Male	8274 (51.3%)	5753 (51.5%)	2521 (50.8%)			0.7 (−1.0 to 2.3)
Preoperative	32.0 (16.5)	30.5 (15.9)	35.4 (17.4)	<0.0001	<0.0001	−5.0 (−5.6 to −4.4)
	31.3 (0–81.3)	31.3 (0–81.3)	37.5 (0–81.3)			
	n=16 131	n=11 172	n=4959			
Postoperative 1 year	57.9 (23.0)	64.5 (20.5)	43.1 (21.4)	<0.0001	<0.0001	21.3 (20.6 to 22.1)
	62.5 (0–100)	68.8 (0–100)	43.8 (0–100)			
	n=16 119	n=11 165	n=4954			

For categorical variables n (%) is presented. For continuous variables Mmean (SD) / Mmedian (Mmin; Mmax) / (95% CI CI for Mmean) / n=is presented. For comparison between groups Fisher´’s Eexact test (lowest 1one-sided p- value multiplied by 2) was used for dichotomous variables and the Mann-Whitney U- test was used for continuous variables.

* Analysis adjusted for age at surgery and pre-operativepreoperative KOOS score.

KOOS, Knee injury and Osteoarthritis Outcome Score; MIC, minimal important change; n, number; QoL, Quality of Life

### Activities at the time of injury

There were significant differences in activities at the time of injury between the no MIC and MIC groups for both the KOOS Sport/Rec and QoL subscales (p<0.0001). However, the differences were small, 0.1%–4.4% (onlinesupplemental appendices tables 1  2).

## Discussion

The main finding of this study was that patients in the no MIC group for both KOOS Sport/Rec and QoL had higher BMI and were younger at the time of surgery. Patients in the no MIC group for Sport/Rec also had a greater proportion of cartilage injuries compared with patients in the MIC group, especially to the lateral femoral condyle. Surprisingly, patients in the MIC group for both the KOOS Sport/Rec and QoL subscales had a higher percentage of meniscal injuries compared with patients in the no MIC group. Although, all statistically significant differences were small and might not be clinically relevant, though results may reframe preoperative and immediate postoperative discussions with patients who have some of these characteristics, particularly for those with chondral injuries.

### Patient demographics

Although it has been previously stated that demographics were not able to predict which patients achieve MIC and not^31^ results from other studies show otherwise.^32^ Our study shows a greater percentage of females in the no MIC group for KOOS Sport/Rec or QoL, and had, increased BMI, were younger age at time of surgery, and had longer time from injury to surgery, compared with patients in the MIC group, although no predictions for the achievement of MIC was made in this study. Previous research has shown that female sex was predictive for lower scores on all KOOS subscales^33^ and that males have significantly higher baseline scores for KOOS QoL.^34^ These results are however somewhat contradictory to results from our study, as there was a greater percentage of females in the no MIC group for KOOS Sport/Rec or QoL and, higher baseline score, which might indicate that MIC is harder to achieve for males than females. Although, males have reported a more positive outlook on their knee related self-efficacy compared with females, which could be the reason a lower percentage of females achieve MIC at 1-year follow-up.^35^ Our study also shows that a lower percentage of patients who are younger at the time of surgery in the no MIC group, and younger age has been associated with higher baseline scores for KOOS QoL.^34^ Higher baseline scores may make it more difficult to achieve MIC after 1-year follow-up. Furthermore, studies show younger patients have higher expectations of their knee function compared with older patients,^36^ which might also be reflected in our results. These results might also imply that younger patients need longer time than 1 year to improve to achieve MIC, or that these younger patients need help adapting to lower expectations of knee function in rehabilitation up to 1-year follow-up. Results from our study also show a greater percentage of patients who had a longer time from injury to surgery did not achieve MIC at 1-year follow-up. Delaying ACLR has also been associated with the risk of development of secondary meniscal injuries,^37 38^ which may further affect perceived knee function.

In addition, a greater proportion of patients treated with the patellar tendon autograft or quadriceps tendon autograft were in the no MIC group compared with patients treated with hamstrings tendon autograft who were in the MIC group. Previous research has shown that patients treated with hamstrings tendon autograft display less knee pain after surgery compared with patients treated with patellar tendon autograft.^39 40^ Furthermore, the hamstrings tendon autograft has reported less comorbidity compared with the patellar-, and quadriceps tendon autografts,^41^ which might also explain clinical differences within these patient groups in the present study.

### Concomitant injuries

Previous research has suggested that concomitant meniscal injuries may predict inferior PROs after ACLR.^42 43^ However, in our analysis, the results suggested a greater proportion of patients in the MIC group despite concomitant meniscal injuries. While the differences between patients in the no MIC and MIC group, with regard to meniscus injuries appear contradictory, this finding may allude to a high prevalence of preoperative knee-related disability. Consequently, patients might achieve MIC by moving from a low score on the KOOS to merely a slightly higher score, and thereby still not have achieved an acceptable knee function according to the patient.

Regarding cartilage injuries, Brophy *et al*^42^ and Cox *et al*^43^ previously highlighted the negative effect of concomitant cartilage injuries on postoperative outcomes after ACLR, which is also seen in the present study as a higher percentage of patients with cartilage injury, especially to the lateral femoral condyle, were in the no MIC group. Another reason for a lower percentage of patients with cartilage injuries achieving the MIC can be due to the advanced surgical procedures with associated postoperative restrictions, resulting in a more restrictive postoperative rehabilitation process.^44^ In addition, patients with cartilage injuries have been reporting greater levels of pain and knee-related symptoms compared with patients without cartilage injuries^45^ which might affect both sports and QoL. Despite results from previous studies,^42 43^ postoperative outcomes might reach MIC values, but do not necessarily relate to a clinical improvement in that is, function. The clinical implication from these results indicates that patients who have a concomitant cartilage injury might not be able to have the same expectations as patients without a cartilage injury, of knee function regarding Sport/Rec up to 1 year after ACLR, and might need longer time to adjust to these disabilities, or might not be as responsive to surgical treatment of the ruptured ACL compared with patients without cartilage injuries. Whether this depends on the complexity of the injury or the treatment, is unknown.

### Limitations

Although this study is registry-based, using a large cohort size, registries have limitations. Due to data configuration, there can be missing data at different follow-ups or for different variables. Furthermore, data from the SNKLR may be limited in terms of generalisability, since data only provide information about the Swedish population with ACLR. Unfortunately, there are no anchor-based questions in the SNKLR and therefore we had no ability to calculate new MIC cut-offs based on the data from SNKLR, instead we used the MIC used by Ingelsrud *et al*.^26^ In addition, the MIC can be calculated in different ways to match different data sets, and MIC might not apply to every unique patient. This means that patients can achieve MIC but do not necessarily equal to acceptable knee function. One year might not be enough to achieve MIC for some patients, as cartilage injuries usually take longer to rehabilitate, and might have persisting knee symptoms compared with an isolated ACLR. However, the MIC used in this study determines changes over time, from baseline to a specific follow-up (6 months, 1 or 2 years), in this case 1 year, and the cut-offs have previously been used.^26^ Furthermore, the MIC values might not have identified all possible MICs after ACLR, as MIC can vary depending on the magnitude of baseline scores for Sports/Rec and QoL.

Using the adjustments, for this study, age at surgery and KOOS preoperatively, might affect the achievement of MIC, as seen for meniscal injuries, as these patients might have a lower preoperative score and therefore a greater chance of achieving MIC. Results from this study can also be difficult to apply in clinic settings since our study mainly studied demographic and surgical characteristics between the no MIC and MIC groups. However, our results may assist surgeons in understanding which patients might benefit from an ACLR and which patients who might not. In line with this, the results from this study might also assist physical therapists in conveying patient expectations for rehabilitation up to 1 year after ACLR.

Finally, the statistically significant differences found in this study were small, thus, significant results might not have been clinically relevant. In addition, there was a large number of missing data in several items when comparing the two groups (MIC vs no MIC), for example, there were 1500 cases of missing BMI data in the No MIC group in the KOOS Sport/Rec. Results from this study should therefore be interpreted with caution.

## Conclusion

Patients who did not achieve the MIC for both KOOS Sport/Rec and QoL had higher BMI, longer time from injury to surgery, and were older at time of surgery compared with patients who did achieve MIC. Patients who did not achieve MIC for KOOS Sport/Rec also had greater rates of concomitant cartilage injuries, especially to the lateral femoral condyle compared with patients who did achieve MIC. The clinical implication is that patients might need a longer time to achieve MIC than 1-year postoperative.

## supplementary material

10.1136/bmjopen-2023-083803 online supplemental file 1

10.1136/bmjopen-2023-083803 online supplemental file 2

## Data Availability

Data are available upon reasonable request.
