# Supplementary material for: Demographic and surgical characteristics in patients who do not achieve minimal important change in the KOOS Sport/Rec and QoL after ACL reconstruction: a comparative study from the Swedish National Knee Ligament Registry
Source: BMJ Open. 2024 Sep 5;14(9):e083803. doi: 10.1136/bmjopen-2023-083803 (PMC11381709; doi:10.1136/bmjopen-2023-083803)
Supplement: online supplemental file 1 [file bmjopen-14-9-s001.pdf]

Appendix table 1: Activity at time of injury for KOOS Sports and recreation

Variable	Total	Achieved MIC	Not achieved MIC	p-value	Adjusted p-value*	Difference between groups Mean (95% CI)
**n**	16 131	11 172	4959			
**Female**	7857 (48.7%)	5419 (48.5%)	2438 (49.2%)	0.45	<.0001	-0.7 (-2.3; 1.0)
**Male**	8274 (51.3%)	5753 (51.5%)	2521 (50.8%)			0.7 (-1.0; 2.3)
**Injury mechanism, n (%)**						
**Alpine/skiing**	2797 (17.4%)	2034 (18.2%)	763 (15.4%)	<.0001	<.0001	
**Pivoting-sport**	10237 (63.6%)	7078 (63.5%)	3159 (63.8%)			
**Non-pivoting sport**	684 (4.2%)	465 (4.2%)	219 (4.4%)			
**Other physical activity**	668 (4.1%)	465 (4.2%)	203 (4.1%)			
**Traffic-related**	257 (1.6%)	151 (1.4%)	106 (2.1%)			
**Other**	1456 (9.0%)	957 (8.6%)	499 (10.1%)			
**Missing**	32	22	10			

CI=Confidence interval, MIC=Minimal important change, n=number
