# Supplementary material for: Demographic and surgical characteristics in patients who do not achieve minimal important change in the KOOS Sport/Rec and QoL after ACL reconstruction: a comparative study from the Swedish National Knee Ligament Registry
Source: BMJ Open. 2024 Sep 5;14(9):e083803. doi: 10.1136/bmjopen-2023-083803 (PMC11381709; doi:10.1136/bmjopen-2023-083803)
Supplement: online supplemental file 2 [file bmjopen-14-9-s002.pdf]

*Appendix table 2: Activity at time of injury for KOOS Quality of Life*

| Variable | Total | Achieved MIC | Not achieve MIC | p-value | Adjusted p-value | Difference between groups Mean (95% CI) |
|---|---|---|---|---|---|---|
| **n** | 16 131 | 10 641 | 5490 | | | |
| **Female** | 7857 (48.7%) | 5240 (49.2%) | 2617 (47.7%) | 0.060 | 0.30 | 1.6 (-0.1; 3.2) |
| **Male** | 8274 (51.3%) | 5401 (50.8%) | 2873 (52.3%) | | | -1.6 (-3.2; 0.1) |
| **Injury mechanism, n (%)** | | | | | | |
| **Alpine/skiing** | 2797 (17.4%) | 1980 (18.7%) | 817 (14.9%) | <.0001 | <.0001 | |
| **Pivoting-sport** | 10237 (63.6%) | 6590 (62.1%) | 3647 (66.5%) | | | |
| **Non-pivoting sport** | 684 (4.2%) | 457 (4.3%) | 227 (4.1%) | | | |
| **Other physical activity** | 668 (4.1%) | 447 (4.2%) | 221 (4.0%) | | | |
| **Traffic-related** | 257 (1.6%) | 147 (1.4%) | 110 (2.0%) | | | |
| **Other** | 1456 (9.0%) | 994 (9.4%) | 462 (8.4%) | | | |
| **Missing** | 32 | 26 | 6 | | | |

CI=Confidence interval, MIC=Minimal important change, n=number
