# Supplementary material for: Demographic and surgical characteristics in patients who do not achieve minimal important change in the KOOS Sport/Rec and QoL after ACL reconstruction: a comparative study from the Swedish National Knee Ligament Registry
Source: BMJ Open. 2024 Sep 5;14(9):e083803. doi: 10.1136/bmjopen-2023-083803 (PMC11381709; doi:10.1136/bmjopen-2023-083803)
Supplement: Supplementary file 3 [file bmjopen-2023-083803.reviewer_comments.pdf]

PEER REVIEW HISTORY

BMJ Open publishes all reviews undertaken for accepted manuscripts. Reviewers are asked to complete a checklist review form and are provided with free text boxes to elaborate on their assessment. These free text comments are reproduced below.

ARTICLE DETAILS

**TITLE (PROVISIONAL)**	Demographic and surgical characteristics in patients who do not achieve minimal important change in the KOOS Sport/Rec and QoL after ACL reconstruction – a comparative study from the Swedish National Knee Ligament Registry
**AUTHORS**	Simonsson, Rebecca; Bittar, Judy; Kaarre, Janina; Zsidai, Bálint; Sansone, Mikael; Piussi, Ramana; Musahl, Volker; Irrgang, James; Samuelsson, Kristian; Senorski, Eric

VERSION 1 - REVIEW

**REVIEWER NAME**	Horii, Manato
**REVIEWER AFFILIATION**	Department of Orthopedic Surgery, Graduate School of Medicine, Chiba University
**REVIEWER CONFLICT OF INTEREST**	None.
**DATE REVIEW RETURNED**	19-Mar-2024

**GENERAL COMMENTS**	Thank you for the opportunity to review this manuscript, entitled "Minimal important change in the KOOS after anterior cruciate ligament reconstruction – a comparative study from the Swedish national knee ligament registry"". In this study, the researchers compared the participants' characteristics and surgical information between the patients who achieved the MIC in the KOOS sports or req at 1 year after ACLR and those who did not. The topic itself is interesting, however, there are several issues. I thought the standard of written English was not acceptable for publication.<br><br>Title:<br>The title may mislead readers. It would be preferable to indicate an interest in KOOS's Sport/Rec or QoL.<br><br>Abstract:<br>L30- this sentence lacks a subject.<br>L35 What is "the XXX" ?<br><br>Introduction:<br>The text in the Introduction is vague and lacks substance. Please specify why there is an interest in the KOOS's Sports/Rec or QoL.<br>L77 Please change "ACL reconstruction" to "ACLR". Multiple sections in the text require similar revisions, for example, L103, L113, and so on.<br><br>Methods:<br>Study design: Please mention within the text that this study is a cross-sectional study.<br>Outcome measure: Please provide the details regarding the surgical factors. For instance, cartilage injury meniscus injury, and so on.

	Results:<br>Cases with other ligament injuries were excluded, yet they appeared in the comparison items. Why is this?<br>The expressions of comparison are not consistent. Please standardize to either "and," "versus," or "vs."<br>Tables: Please ensure the number of decimal places is consistent for each item.<br><br>Discussion:<br>L234-236 Please remove the sentence. This is not necessary.<br>Limitations: Please mention that there were missing data in several items when comparing the two groups. Notably, there were 1,500 cases of missing BMI data in the No MIC group in KOOS Sports/Rec.

**REVIEWER NAME**	Arumugam, Ashokan
**REVIEWER AFFILIATION**	University of Sharjah, Department of Physiotherapy
**REVIEWER CONFLICT OF INTEREST**	None
**DATE REVIEW RETURNED**	22-Mar-2024

**GENERAL COMMENTS**	I would like to applaud the efforts of the authors for conducting a study investigating differences in demographic and surgical characteristics between patients who do and do not achieve MIC in the KOOS Sports and Recreation and Quality of Life subscales one year after anterior cruciate ligament reconstruction. Please find below my comments and suggestions to improve the content and clarity of the manuscript.<br><br>L34 setting must be changed as appropriate.<br><br>L49 ....reframe “discussions”? Perhaps an alternative term is required (e.g., management strategies or anything else?).<br><br>L74 Why Knee injury and Osteoarthritis Outcome Score (KOOS) subscales chosen specifically but not the other PRO scales relevant to assessing knee outcomes in people with ACL injuries? This needs justification.<br><br>L75-76 Are MIC and MCID the same? As you have added MCID as a keyword, it’s worth explaining these terms in the introduction. Please define these terms as appropriate for the readers to understand them explicitly.<br><br>L90 The data were obtained only up to May 2019 from the Swedish national knee ligament registry (SNKLR). Was there a lack of updates on the relevant data on the registry after that?<br><br>L100 Any other PRO data available in the registry?<br><br>Please refer to reference 16 quoted in your manuscript. In the Ingelsrund et al. study, they seem to have included only young adults. However, in your study, you chose individuals ≥15 years of age? Is there a specific reason for choosing adolescents in addition to adults in your study? What is the maximum age of participants included in your study? Were older adults included in your study?

	Ingelsrund et al. reported, “The MIC for improvement was calculated with anchor-based approaches using the predictive modeling method adjusted for the proportion of improved patients, the mean change method, and the receiver operating characteristic (ROC) method.”<br><br>You have used data from Ingelsrund et al. based on the data of participants from the Norwegian Knee Ligament Registry. Would it be possible to calculate MIC values for your participants from the SNKLR and then use them to answer your research questions? This would be appropriate for your study's aims.<br><br>L121 Why was the smoking status of participants retrieved from the registry? It has been included as a participant characteristic in the tables. Is there any relevance to MIC for this variable?<br><br>Once the methodological clarifications are available for the comments above, further review of the manuscript will be meaningful.<br><br>The authors must thoroughly proofread the manuscript for English language errors.<br><br>Thank you for the opportunity to review the manuscript.

VERSION 1 – AUTHOR RESPONSE

Reviewer: 1 Dr. Manato Horii, Department of Orthopedic Surgery, Graduate School of Medicine, Chiba University

Comments to the Author: Thank you for the opportunity to review this manuscript, entitled "Minimal important change in the KOOS after anterior cruciate ligament reconstruction – a comparative study from the Swedish national knee ligament registry"". In this study, the researchers compared the participants’ characteristics and surgical information between the patients who achieved the MIC in the KOOS sports or req at 1 year after ACLR and those who did not. The topic itself is interesting, however, there are several issues. I thought the standard of written. English was not acceptable for publication.

Dear Dr. Horii,

Thank you for taking the time to review our article. We have carefully considered all your comments as suggested in order to improve our manuscript for publication. We have asked a language consultant to go through the manuscript in order to revise the language, to make it acceptable for publication. Please see answers to comments below.

Title:

The title may mislead readers. It would be preferable to indicate an interest in KOOS's Sport/Rec or QoL.

Answer: Thank you for this comment. We have rephrased the title to clarify the use of KOOS subscales Sport/Rec and QoL. Amendments to lines 1-3.

“Minimal important change in the KOOS Sport/Rec and QoL after anterior cruciate ligament reconstruction – a comparative study from the Swedish national knee ligament registry”

Abstract:

L30- this sentence lacks a subject.

Answer: A subject has been added to the sentence. Amendments to likes 30-33:

“This study aimed to compare demographic- and surgical characteristics between patients who do and do not achieve MIC in the KOOS Sports and Recreation (Sport/Rec) and Quality of Life (QoL) subscales 1 year after anterior cruciate ligament reconstruction (ACLR).”

L35 What is “the XXX” ?

Answer: XXX was blinded for the “Swedish National Knee Ligament Registry”. This has now been changed. See amendments to lines 35-37:

“The MIC for the KOOS Sport/Rec subscale was ≥12.1 and ≥18.3 for the KOOS QoL subscale from before surgery to 1-year follow-up using data from the Swedish National Knee Ligament Registry.”

Introduction:

The text in the Introduction is vague and lacks substance. Please specify why there is an interest in the KOOS's Sports/Rec or QoL.

Answer: Thank you for this comment. We have extended the introduction section to answer this comment. Amendment to lines 69-75:

“Thus, patient-reported outcomes (PROs) have been frequently used to assess the patient’s perception of their knee function and to evaluate the effects and outcomes of surgical treatment of an ACL injury (2-4). One PRO commonly used in patients treated with ACLR is the Knee injury and Osteoarthritis Outcome Score (KOOS), which contain 5 subscales which evaluate pain, symptoms, activities of daily living (ADL), sport and recreation and, quality of life (5). However, only the KOOS subscales sports and recreation, and quality of life fulfilled the criteria of unidimentionality for patients treated with ACLR (6).”

We have also added four references to this section to support this statement:

2. Muller B, Yabroudi MA, Lynch A, et al. Defining Thresholds for the Patient Acceptable Symptom State for the IKDC Subjective Knee Form and KOOS for Patients Who Underwent ACL Reconstruction. Am J Sports Med 2016;44(11):2820-26. doi: 10.1177/0363546516652888 [published Online First: 2016/07/31

3. Nwachukwu BU, Adjei J, Rauck RC, et al. How Much Do Psychological Factors Affect Lack of Return to Play After Anterior Cruciate Ligament Reconstruction? A Systematic Review. Orthop J Sports Med 2019;7(5):2325967119845313. doi: 10.1177/2325967119845313 [published Online First: 2019/06/18]

4. Mai HT, Chun DS, Schneider AD, et al. Performance-Based Outcomes After Anterior Cruciate Ligament Reconstruction in Professional Athletes Differ Between Sports. Am J Sports Med 2017;45(10):2226-32. doi: 10.1177/0363546517704834 [published Online First: 20170516]

5. Roos EM, Lohmander LS. The Knee injury and Osteoarthritis Outcome Score (KOOS): from joint injury to osteoarthritis. Health Qual Life Outcomes 2003;1:64. doi: 10.1186/1477-7525-1-64 [published Online First: 2003/11/14]

6. Comins J, Brodersen J, Krogsgaard M, Beyer N. Rasch analysis of the Knee injury and Osteoarthritis Outcome Score (KOOS): a statistical re-evaluation. Scand J Med Sci Sports 2008;18(3):336-45. doi: 10.1111/j.1600-0838.2007.00724.x

L77 Please change “ACL reconstruction” to “ACLR”. Multiple sections in the text require similar revisions, for example, L103, L113, and so on.

Answer: Thank you for this comment. We have changed “ACL reconstruction” to ACLR. Amendments below:

Lines 87-89: “Although numerous factors have been reported to affect outcomes after ACLR i including graft failure, reinjury, and inferior PROs, factors associated with clinically relevant changes in PROs remain to a large extent undefined.”

Lines 93-96: “In addition, a deeper understanding of which patients who are not likely to experience clinical improvement after ACLR and do thereby not respond to ACLR as a treatment is of evident need in order for patients to receive the best possible treatment after ACL injury.”

Lines 111-114: “The SNKLR aims to promote superior treatment for individuals with ACL injuries by providing hospitals and clinics with accurate feedback with regard to surgical technique and outcomes after ACLR, and to determine prognostic factors for successful and poor outcomes.”

Lines 120-121: “Patients are asked to respond to questionnaires at 1-, 2-, 5- and 10-year follow-ups after ACLR.”

Lines 132-133: “Patients eligible for inclusion were ≥15 years old, had undergone a primary ACLR and had KOOS data for the Sport/Rec and QoL subscales before surgery and at 1 year follow-up.”

Lines 287-289: “Delaying ACLR has also been associated with the risk of development of secondary meniscal injuries, which may further affect perceived knee function.”

Lines 334-336: “Furthermore, the MIC values might not have identified all possible MICs after ACLR, as MIC can vary depending on the magnitude of baseline scores for Sports/Rec and QoL.”

Methods:

Study design: Please mention within the text that this study is a cross-sectional study.

Answer: Thank you for this comment. This study was conducted as a comparative study based on registry data from the Swedish National Knee Ligament Registry. The comparison is cross-sectional but the grouping is based on pre- and post operative data. However, we have clarified that the present study is a comparative registry study on lines 103-108:

“This study was conducted as a comparative registry study, where data was analysed cross sectionally and was reported according to the Reporting of studies Conducted using Observational

Routinely-collected Data (RECORD) statement, which is an extension of the Strengthening the Reporting of Observational Studies in Epidemiology (STROBE) guidelines with data from the Swedish national knee ligament registry (SNKLR) during the period January 2005 - May 2019."

Outcome measure: Please provide the details regarding the surgical factors. For instance, cartilage injury meniscus injury, and so on.

Answer: Thank you for your comment. We have made amendments to lines 147-150:

"The main outcome of interest in this study was differences in demographical (e.g., age, sex, BMI, activity at time of injury) and surgical factors (e.g., type of graft, cartilage injuries, meniscal injuries, collateral ligament injures) between patients who achieved the MIC and patients who did not achieve the MIC, for the KOOS subscales Sport/Rec and QoL, between pre-operative ratings and 1 year after ACLR."

Results:

Cases with other ligament injuries were excluded, yet they appeared in the comparison items. Why is this?

Answer: Thank you for this comment. The ligament injuries that were excluded were the grade 3 medial collateral ligament ruptures that required surgically reconstruction. We have tried to make this clear in the sentence which addresses exclusion criteria on lines 134-137:

"Patients with previous knee surgery, associated fracture to the patella, femur, tibia or fibula, or concomitant nerve, vascular or additional injuries to any other knee ligaments, such as the posterior cruciate ligament (PCL), or had a surgically reconstructed medial or lateral collateral ligament (MCL, LCL) tear were excluded from this study."

The MCL and LCL injuries that appear in the comparison items are the injuries that did not require surgical reconstruction. We hope we made this clearer by highlighting the specification in the section addressing exclusions. If this needs further clarification, please let us know.

The expressions of comparison are not consistent. Please standardize to either "and," "versus," or "vs."

Answer: We have made amendments throughout the text and provided each sentence addressing comparisons with "versus". See amendments below:

Lines 198-200: "The no MIC group had a significantly greater BMI compared to the MIC group (24.8 ± 3.5 versus 24.6 ± 3.3 respectively, p<.0001)."

Lines 200-203: "A greater proportion of patients in the no MIC group were younger at the time of surgery (28.5 ±10.3 versus 29.1 ±10.8 years, respectively, p=0.0002) and had a longer time from injury to surgery (22.0 ± 38.5 versus 19.3 ± 36.6 months, respectively, p=0.0002) compared to patients in the MIC group."

Lines 211-214: "Patients in the no MIC group had a greater rate of concomitant cartilage injuries (27.7% versus 26.7% respectively, p<0.0001) especially located on the lateral femoral condyle

(22.7% versus 19.4% respectively, p=0.0009) or in the presence of a multicartilage injury (27.7% versus 26.5%, p<0.0001) compared to patients in the MIC group."

Lines 218-220: "Patients in the no MIC group also had lowered their scores post-operatively and had significantly lower scores post-operative compared to the MIC group (48.2 ± 26.5 versus 73.0 ± 20.9 respectively, p<0.0001)."

Lines 223-225: "Patients in the no MIC group for KOOS QoL had greater BMI (24.7 ± 3.5 versus 24.6 ± 3.2 respectively, p=0.0001) and were younger at the time of surgery compared to patients in the MIC group (27.4 ± 9.8 versus 29.7 ± 11.0, respectively, p<.0001)."

Lines 230-234: "Table 3 presents patients in the no MIC group who had greater rate of concomitant cartilage injuries to the lateral femoral condyle (23.3% versus 19.0%, p=0.0048), however, lower rate of general cartilage injury (26.1% versus 27.5%, respectively, p=0.0024), to the medial patella (29.9% versus. 20.5%, p=0.027) and multicartilage injuries (26.0% versus 27.3%, p=0.0021) compared to patients in the MIC group."

Lines 239-242: "Both patients in the no MIC and MIC group had increased their scores postoperatively, although patients in the no MIC group had significantly lower scores compared to the patients in the MIC group overall (43.1 ± 21.4 versus 64.5 ± 20.5 respectively, p<0.0001)."

Tables: Please ensure the number of decimal places is consistent for each item.

Answer: We have now changed the number of decimals to Table 1, BMI, age and Time from injury to surgery.

Table 1, 3 and 4: We have also changed the non-significant p-values for standardization. However, p-values that were significant and could not be changed to an equal number of decimals as the non-significant values were chosen to stay the actual number of decimals for accuracy.

Discussion:

L234-236 Please remove the sentence. This is not necessary.

Answer: We have removed this sentence from the manuscript in accordance with this comment.

Limitations: Please mention that there were missing data in several items when comparing the two groups. Notably, there were 1,500 cases of missing BMI data in the No MIC group in KOOS Sports/Rec.

Answer: Thank you for this comment. We have added a sentence on this topic in the limitations section to lines 346-348:

"In addition, there were a large number of missing data in several items when comparing the two groups (MIC versus No MIC), e.g., there were 1.500 cases of missing BMI data in the No MIC group in the KOOS Sport/Rec."

Reviewer: 2 Dr. Ashokan Arumugam, University of Sharjah

Comments to the Author: I would like to applaud the efforts of the authors for conducting a study investigating differences in demographic and surgical characteristics between patients who do and do not achieve MIC in the KOOS Sports and Recreation and Quality of Life subscales one year after anterior cruciate ligament reconstruction. Please find below my comments and suggestions to improve the content and clarity of the manuscript.

Dear Dr. Arumugam,

We greatly appreciate your time and effort to review our manuscript and provide us with comments to improve the outcome of this manuscript prior to publication. We have answered all your comments and made amendments to specific lines for respective comments. Please find our answers below.

L34 setting must be changed as appropriate.

Answer: Please forgive us for not entirely understanding this comment. We have written “Swedish National Knee Ligament Registry” instead of using the masked version “XXX”, if this comment was referred to that. If not, please let us know how to revise according to this comment. Thank you. Amendment on lines 35-37:

“The MIC for the KOOS Sport/Rec subscale was ≥12.1 and ≥18.3 for the KOOS QoL subscale from before surgery to 1-year follow-up using data from the Swedish National Knee Ligament Registry.”

L49 ....reframe “discussions”? Perhaps an alternative term is required (e.g., management strategies or anything else?).

Answer: Thank you for this comment, we have chosen to rephrase “discussion” to “management strategies” according to this comment. Amendment to lines 51-52:

“Although differences were small, they may reframe management strategies with patients who have these characteristics.”

L74 Why Knee injury and Osteoarthritis Outcome Score (KOOS) subscales chosen specifically but not the other PRO scales relevant to assessing knee outcomes in people with ACL injuries? This needs justification.

Answer: We chose to use only the KOOS subscales sports and recreation and quality of life because of the Rasch analysis made 2008 by Comins et al. where the authors determined that the subscales sports and recreation and quality of life were the only two subscales in the KOOS questionnaire that fulfilled the criteria of unidimentionality for patients treated with ACLR. A clarification has been made to lines 74-75:

“However, only the KOOS subscales sports and recreation, and quality of life fulfilled the criteria of unidirectionality for patients treated with ACLR (6).”

We also added a reference to support this statement,

6. Comins J, Brodersen J, Krogsgaard M, Beyer N. Rasch analysis of the Knee injury and Osteoarthritis Outcome Score (KOOS): a statistical re-evaluation. Scand J Med Sci Sports 2008;18(3):336-45. doi: 10.1111/j.1600-0838.2007.00724.x

L75-76 Are MIC and MCID the same? As you have added MCID as a keyword, it's worth explaining these terms in the introduction. Please define these terms as appropriate for the readers to understand them explicitly.

Answer: Thank you for this comment. We have tried to clarify this in the introduction section and have added a couple of sentences on the topic. Amendments to line 75-85:

"Despite a wide range of studies that report outcomes for patients with an ACL injury with PROs,(7) interpretation of the scores are still difficult for the ACL research. To aid clinicians to interpret scores from PROs, several concepts can be calculated, e.g., the minimum clinically important difference (MCID) and the minimal important change (MIC). The MCID represents the smallest improvement considered worthwhile by a patient.(8) The MIC instead reflects the smallest measured change in score that patients perceive as important.(9) When it comes to clinically meaningful effects, the terms MIC and MCID are used, not always consistently, and often in ways that make interpretation difficult.(10) The MIC and the MCID are fundamentally different.(10) As semantically described, a change (MIC) occurs within one group, while a difference (MCID) occurs between groups. "

We have also added four new references to support those statements;

7. Gagnier JJ, Shen Y, Huang H. Psychometric Properties of Patient-Reported Outcome Measures for Use in Patients with Anterior Cruciate Ligament Injuries: A Systematic Review. JBJS Rev 2018;6(4):e5. doi: 10.2106/jbjs.Rvw.17.00114

8. Copay AG, Subach BR, Glassman SD, et al. Understanding the minimum clinically important difference: a review of concepts and methods. Spine J 2007;7(5):541-6. doi: 10.1016/j.spinee.2007.01.008

9. Terwee CB, Peipert JD, Chapman R, et al. Minimal important change (MIC): a conceptual clarification and systematic review of MIC estimates of PROMIS measures. Quality of Life Research 2021;30(10):2729-54. doi: 10.1007/s11136-021-02925-y

10. Kamper SJ. Interpreting Outcomes 3-Clinical Meaningfulness: Linking Evidence to Practice. J Orthop Sports Phys Ther 2019;49(9):677-78. doi: 10.2519/jospt.2019.0705

L90 The data were obtained only up to May 2019 from the Swedish national knee ligament registry (SNKLR). Was there a lack of updates on the relevant data on the registry after that?

Answer: Thank you for this comment. Data was extracted until 2020-12-31, and due to the fact that the follow-up was set to 1 year, data was used between 2005-2019. Every study of which is conducted from the SNKLR needs to be approved from the board committee and our application was accepted for the database with data between 2005-2020, since the application was performed in 2020.

L100 Any other PRO data available in the registry?

Answer: Yes, the other PRO available in the registry are the EuroQoL – 5 Dimentions. A clarification about the PROs has been added to lines 117-119:

“The secondary source contains information registered by the patient including PROs, including the KOOS and EuroQoL – 5 Dimention (EQ-5D). Only the KOOS was used in this study. “

Please refer to reference 16 quoted in your manuscript. In the Ingelsrund et al. study, they seem to have included only young adults. However, in your study, you chose individuals ≥15 years of age? Is there a specific reason for choosing adolescents in addition to adults in your study? What is the maximum age of participants included in your study? Were older adults included in your study?

Answer: Our study obtained ethical approval for inclusion of patients aged ≥15 years. We aimed to cover the general population of patients who suffer an ACL injury in Sweden, and thereby wanted a broad age spectrum. We did not have a maximum age limit, and the oldest participant in our study was 74 years old. Demographics of age distribution can be found in table 1 and table 3. We have not made any amendments according to this comment.

Ingelsrund et al. reported, “The MIC for improvement was calculated with anchor-based approaches using the predictive modeling method adjusted for the proportion of improved patients, the mean change method, and the receiver operating characteristic (ROC) method.”

Answer: Thank you for this comment. Ingelsrud et al. stated three different methods in their article, and we chose to use the anchor-based method and predictive modeling for this study as this method anchors the PRO change scores to an external measure of important change. We have a sentence on this topic in the methods section under “Outcome measures” on lines 154-159:

“The MIC refers to the change from baseline to a specific follow-up timepoint for a given PRO that is perceived as being important by a patient. The MIC used in this study was calculated using an anchor-based method and predictive modeling, as this method anchors the PRO change score to an external measure of important change. Furthermore, the anchor-based method has the best accuracy in predictive modelling and does not easily overestimate the MIC.”

You have used data from Ingelsrund et al. based on the data of participants from the Norwegian Knee Ligament Registry. Would it be possible to calculate MIC values for your participants from the SNKLR and then use them to answer your research questions? This would be appropriate for your study's aims.

Answer: Thank you for this comment. Unfortunately, we do not have the anchor-based question in the SNKLR and are therefore not able to calculate new MIC based on the data from SNKLR, thereby we used the MIC used by Ingelsrud et al.

L121 Why was the smoking status of participants retrieved from the registry? It has been included as a participant characteristic in the tables. Is there any relevance to MIC for this variable?

Answer: Thank you for this comment. We have discussed this among the research group and have decided that the smoking status is not relevant to the scope of this study and have thereby chosen to remove smoking status from the participant characteristics in the tables.

Once the methodological clarifications are available for the comments above, further review of the manuscript will be meaningful.

Answer: Thank you for your comments. We are looking forward to your additional comments after our revision.

The authors must thoroughly proofread the manuscript for English language errors.

Answer: We have asked a language consultant to go through the manuscript in order to revise the language, to make it acceptable for publication.

Thank you for the opportunity to review the manuscript.

Answer: Thank you for taking the time to review our manuscript for improvements for publication. We have appreciated all comments and are looking forward to your additional comments after our clarification and revisions of the manuscript.

Reviewer: 1

Competing interests of Reviewer: None.

Reviewer: 2

Competing interests of Reviewer: None

VERSION 2 – REVIEW

**REVIEWER NAME**	Arumugam, Ashokan
**REVIEWER AFFILIATION**	University of Sharjah, Department of Physiotherapy
**REVIEWER CONFLICT OF INTEREST**	None.
**DATE REVIEW RETURNED**	26-Jun-2024

**GENERAL COMMENTS**	Many thanks to the authors for revising the manuscript based on the reviewer's comments. Well done!<br><br>Please find below some more minor comments:<br><br>Please add a reference for – "As semantically described, a change (MIC) occurs within one group, while a difference (MCID) occurs between groups."<br><br>Please add this as a limitation of the study "Unfortunately, we do not have the

	anchor-based question in the SNKLR and are therefore not able to calculate new MIC based on the data from SNKLR, thereby we used the MIC used by Ingelsrud et al." – paraphrase as required.<br><br>Add this under the discussion section - In the Ingelsrund et al. study, they seem to have included only young adults. However, in your study, you chose<br>individuals ≥15 years of age? Explain the reasons for these differences.

VERSION 2 – AUTHOR RESPONSE

Reviewer: 2

Dr. Ashokan Arumugam, University of Sharjah

Comments to the Author:

Many thanks to the authors for revising the manuscript based on the reviewer's comments. Well done!

Dear Dr. Arumugam, thank you for your valuable input and suggestions for this article. We appreciate your time and effort for improving this article. Please find our response to each comment below.

Please find below some more minor comments:

Please add a reference for – "As semantically described, a change (MIC) occurs within one group, while a difference (MCID) occurs between groups."

Answer: Thank you for this comment. We have added a reference to support the statement in this sentence. Reference has been added to line 86 and is referred to as reference 11.

11. Beaton DE, Bombardier C, Katz JN, et al. Looking for important change/differences in studies of responsiveness. OMERACT MCID Working Group. Outcome Measures in Rheumatology. Minimal Clinically Important Difference. The Journal of Rheumatology 2001;28(2):400.

Please add this as a limitation of the study "Unfortunately, we do not have the

anchor-based question in the SNKLR and are therefore not able to calculate new MIC based on the data from SNKLR, thereby we used the MIC used by Ingelsrud et al." – paraphrase as required.

Answer: The suggested sentence has been added to the limitations section to lines 325-327. The sentence now read:

“Unfortunately, there are no anchor-based questions in the SNKLR and therefore we had no ability to calculate new MIC cut-offs based on the data from SNKLR, instead we used the MIC used by Ingelsrud et al.(26).”

26. Ingelsrud LH, Terwee CB, Terluin B, et al. Meaningful Change Scores in the Knee Injury and Osteoarthritis Outcome Score in Patients Undergoing Anterior Cruciate Ligament Reconstruction. Am J Sports Med 2018;46(5):1120-28. doi: 10.1177/0363546518759543

Add this under the discussion section - In the Ingelsrund et al. study, they seem to have included only young adults. However, in your study, you chose individuals ≥15 years of age? Explain the reasons for these differences.

Answer: Thank you for this comment. In the Ingelsrud et al. study, a questionnaire was sent out to “…. 1197 randomly selected patients who were at 3 different follow-up time points after surgery: 397 at 6 months (range, 5-7 months), 400 at 12 months (range, 10-14 months), and 400 at 24 months (range, 20-28 months) postoperatively. Patients who had undergone bilateral or revision ACL reconstruction were excluded. Individual patients’ preoperative data were retrieved from the NKLR database.” Here stated without age limitations.

Mean age Ingelsrud et al.

Mean age in our study

Since Ingelsrud et al. did not state an age limitation, the choice of using young adults are predominantly due to the questionnaire responders and not to the specific age. Thereby, we do not believe that the age question has to be addressed according to the chosen cut-offs used for the MIC in KOOS. No amendments have been made.

Reviewer: 2

Competing interests of Reviewer: None.
